# Influence of Different Post-Plasma Treatment Storage Conditions on the Shear Bond Strength of Veneering Porcelain to Zirconia

**DOI:** 10.3390/ma9010043

**Published:** 2016-01-12

**Authors:** Mun-Hwan Lee, Bong Ki Min, Jun Sik Son, Tae-Yub Kwon

**Affiliations:** 1 Department of Medical & Biological Engineering, Graduate School, Kyungpook National University, 2-188-1 Samduk-dong, Jung-gu, Daegu 700-412, Korea; leemunhwan@knu.ac.kr; 2 Center for Research Facilities, Yeungnam University, 214-1 Dae-dong, Gyeongsan 712-749, Korea; bkmin@ynu.ac.kr; 3 Korea Textile Development Institute, 1083 Jungri-dong, Seo-gu, Daegu 703-712, Korea; sonjk1@empas.com; 4 Department of Dental Biomaterials, School of Dentistry, Kyungpook National University, 2-188-1 Samduk-dong, Jung-gu, Daegu 700-412, Korea

**Keywords:** zirconia, veneering porcelain, bond strength, plasma treatment, storage condition

## Abstract

This *in vitro* study investigated whether different storage conditions of plasma-treated zirconia specimens affect the shear bond strength of veneering porcelain. Zirconia plates were treated with a non-thermal atmospheric argon plasma (200 W, 600 s). Porcelain veneering (2.38 mm in diameter) was performed immediately (P-I) or after 24 h storage in water (P-W) or air (P-A) on the treated surfaces (*n* = 10). Untreated plates were used as the control. Each group was further divided into two subgroups according to the application of a ceramic liner. All veneered specimens underwent a shear bond strength (SBS) test. In the X-ray photoelectron spectroscopy (XPS) analysis, the oxygen/carbon ratios of the plasma-treated groups increased in comparison with those of the control group. When a liner was not used, the three plasma-treated groups showed significantly higher SBS values than the control group (*p* < 0.001), although group P-A exhibited a significantly lower value than the other two groups (*p* < 0.05). The liner application negatively affected bonding in groups P-I and P-W (*p* < 0.05). When the veneering step was delayed after plasma treatment of zirconia, storage of the specimens in water was effective in maintaining the cleaned surfaces for optimal bonding with the veneering porcelain.

## 1. Introduction

Recently, zirconia, specifically yttria-stabilized tetragonal zirconia polycrystal (Y-TZP), has become favored as a core material for crowns and fixed dental prostheses (FDPs) mainly due to its improved fracture resistance by transformation toughening [1,2]. The zirconia-based core structure is veneered with zirconia veneering ceramic, which generally consists of an amorphous and glassy silica matrix embedded in varying amounts of feldspar and leucite crystals [3].

In zirconia-based restorations, however, chipping and/or delamination of veneering ceramic has been reported at higher rates compared with metal-ceramic systems [4,5]. Ceramic chipping caused by an inherent weakness of the veneering porcelain is one of the most common failures of zirconia-based restorations [6,7,8]. Another clinical problem is delamination at the zirconia-veneer interface, probably caused by the weak interfacial adhesion and large difference in mechanical properties of the zirconia and veneer materials [7,9]. Thus, metal frameworks veneered with ceramics still represent the standard for ceramic FDPs [5]. Enhancing bonding between zirconia and veneering porcelain is required to ensure the long-term clinical success of zirconia-based restorations.

Various surface conditioning methods of the zirconia substructure have been researched to achieve enhanced zirconia/porcelain bonding. Among these methods, airborne-particle abrasion and ceramic liner application are the most commonly studied [7]. Air-abrasion of zirconia with alumina particles may increase the surface roughness and provide undercuts for bonding with veneering porcelain [5]. Some manufacturers recommend the application of a liner on the zirconia core surface to improve bonding and to mask the opaqueness of the zirconia [7]. However, the effect of the liner on the bond strength of the zirconia-veneer interface remains controversial. Kim *et al.* [5] suggested that the application of a liner increased the possibility of interfacial failure of the veneering ceramic from the zirconia core and that airborne-particle abrasion would be more useful than a liner application. Moreover, Wang *et al.* [7] showed that that the application of a liner before veneering reduces the bond strength of the interface.

The chemical composition of commercial ceramic liners varies depending on the manufacturer; however, the primary component is silica, indicating a similar composition to veneering porcelain [5,7]. Thus, the properties of a ceramic liner resemble those of veneering porcelain, and a stronger bond is expected at the porcelain/liner interface than at the liner/zirconia interface [7]. The wetting behavior is of primary importance for good adhesion of veneering porcelain or ceramic liner onto the zirconia substructure [10]. The wetting behavior is dependent not only on the porcelain or liner composition but also on the morphology and surface energy of the zirconia material [11].

Non-thermal plasmas are partially ionized “cold” gases that contain highly reactive particles [12]. Due to the relatively mild, non-destructive character of the gas phase, non-thermal plasma surface treatment effectively alters the surface characteristics, leaving the bulk properties of the materials un-altered after treatment [13]. Plasma surface treatment is generally used for cleaning and surface activation, which raise the “wettability” and surface energy of the substrate and lead to an improvement of the bonding properties [14,15]. A recent study [4] showed that argon plasma treatment improved bonding between zirconia and veneering porcelain. Although such plasma cleaning may be simple and useful for enhancing zirconia-veneer bonding, plasma-cleaned zirconia surfaces may be easily re-contaminated once exposed to air [16]. In a dental laboratory, the porcelain veneering step may be delayed for various reasons, allowing re-contamination, which may affect the bonding of veneered porcelain to the plasma-treated zirconia surfaces. For the practical application of this technology, the surface properties of plasma-treated substrates during storage should be maintained [17].

**Figure 1:**
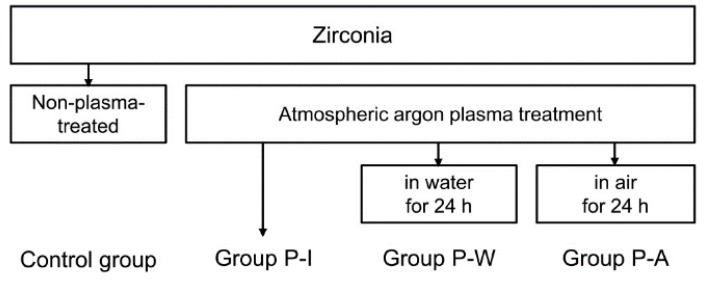
Study design for one untreated (control) and three plasma-treated groups. In the shear bond strength test, each group was further divided into two subgroups according to the use of a liner prior to veneering.

The purpose of this *in vitro* study was to investigate the influence of different post-plasma treatment storage conditions on the shear bond strength (SBS) between zirconia and veneered porcelain. To investigate the combined effect of the plasma and liner, specimens coated with a liner prior to plasma treatment were also prepared (Figure 1). The null hypothesis tested was that different storage conditions after plasma treatment do not affect the SBS between zirconia and veneered porcelain.

## 2. Results

### 2.1. Scanning Electron Microscopy and Atomic Force Microscopy

**Figure 2:**
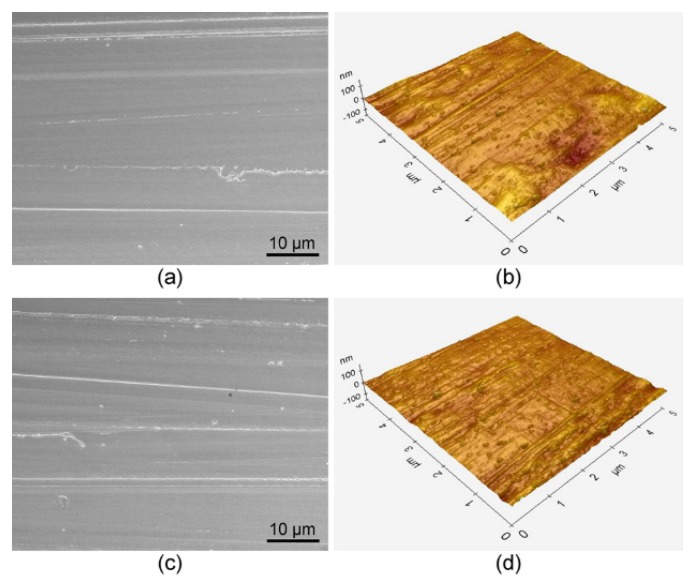
Scanning electron microscopy (SEM) (left, magnification: 2000×, bar = 10 μm) and atomic force microscopy (AFM) (right, 5 μm × 5 μm) images of the zirconia surfaces: (**a**,**b**) untreated; (**c**,**d**) plasma-treated.

Representative scanning electron microscopy (SEM) and atomic force microscopy (AFM) images before and after the plasma treatment are shown in Figure 2. No morphological changes were apparent in the images following treatment. In addition, there was no significant difference in average surface roughness (*R*_a_) between the untreated and the plasma-treated zirconia surfaces (*p* = 0.828).

### 2.2. Water Contact Angles

**Figure 3:**
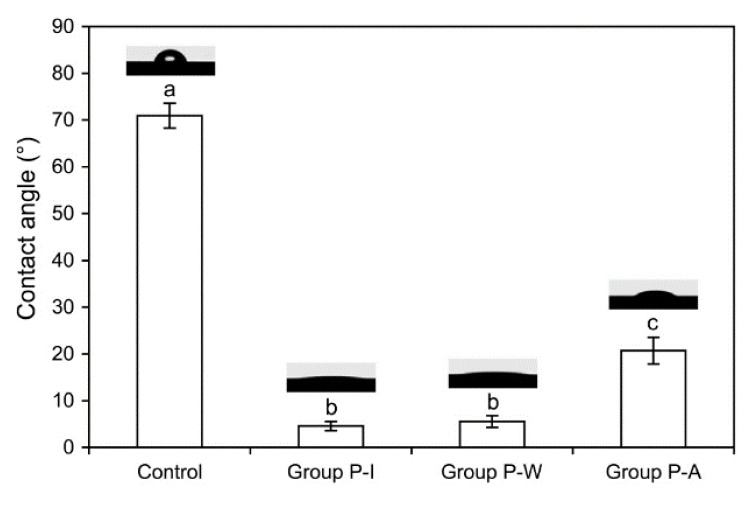
Contact angles of water droplets on the zirconia surfaces (*n* = 6). Different lowercase letters indicate significant differences (*p* < 0.05).

Figure 3 shows the water contact angle (CA) values for the four experimental groups. The control (untreated) group showed the highest CA among the groups, the difference being statistically significant (*p* < 0.001). The plasma treatment significantly lowered the CA values in comparison with the control group, although group P-A showed a significantly higher value than groups P-I and P-W (*p* < 0.001).

### 2.3. X-ray Photoelectron Spectroscopy

**Figure 4:**
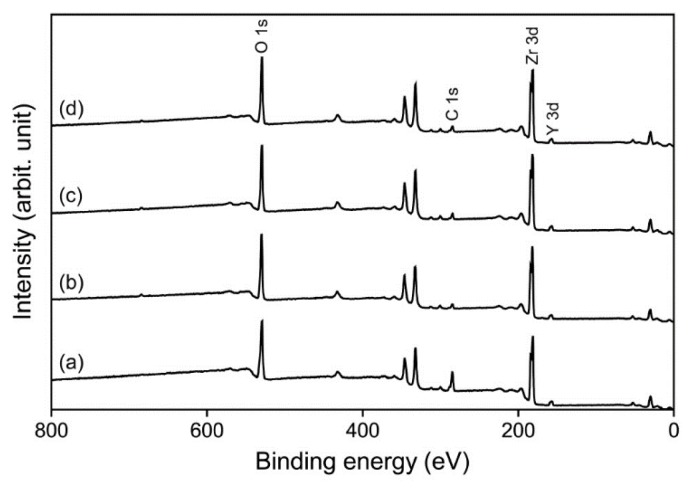
Survey X-ray photoelectron spectroscopy (XPS) spectra for untreated (**a**) and plasma-treated (**b**–**d**) zirconia surfaces: groups P-I (b); P-W (c); and P-A (d).

**Figure 5:**
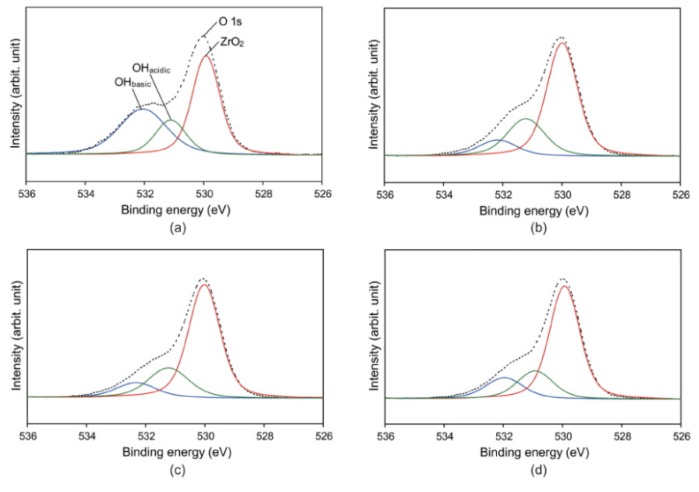
High-resolution X-ray photoelectron spectroscopy (XPS) spectra of O 1s region of untreated (**a**) and plasma-treated (**b**–**d**) zirconia surfaces: groups P-I (b); P-W (c); and P-A (d). Deconvolution of O 1s high resolution spectrum resulted in three components: basic hydroxyl groups at 532.6 eV, acidic hydroxyl groups at 531.8 eV, and ZrO_2_ at 530.2 eV.

The results of the X-ray photoelectron spectroscopy (XPS) analysis are shown in Figure 4 and Figure 5 and Table 1. The oxygen/carbon (O/C) ratios of the plasma-treated groups were considerably increased compared to that of the control group due to an increase in oxygen content (at %) and a decrease in carbon content. When the O 1s peak was deconvoluted into three components (ZrO_2_ and acidic/basic hydroxyl groups), an increased oxygen content in the plasma-treated groups was found. The amount of basic hydroxyl groups was substantially decreased in the plasma-treated groups when compared to the control group, being lower in groups P-I and P-W than in group P-A.

**Table 1:** Atomic percent of each element and oxygen/carbon (O/C) ratio for each group, calculated from the survey X-ray photoelectron spectroscopy (XPS) spectra (Figure 4).

Group	Zr 3d	O 1s	Y 3d	C 1s	O/C
Control	15.05	48.32	1.32	35.31	1.37
P-I	20.73	64.93	1.71	12.63	5.14
P-W	22.31	62.04	1.66	13.99	4.43
P-A	22.11	61.23	1.65	15.01	4.08

### 2.4. Shear Bond Strength and Failure Pattern

Table 2 shows the shear bond strength (SBS) value for each experimental group. Two-way analysis of variance (ANOVA) showed statistically significant differences based on the storage condition (including control) and for the interaction between the two main effects (*p* < 0.001). Within the no-liner subgroups, the lowest SBS was obtained in the control group, the difference being statistically significant (*p* < 0.001). Of the plasma-treated groups, groups P-I and P-W, which were statistically similar to each other (*p* = 0.311), exhibited significantly higher SBS values than group P-A (*p* < 0.05). On the other hand, there were no significant differences in the SBS values among the four liner subgroups (*p* = 0.742). Within the control (untreated) group, liner application significantly increased the SBS (*p* < 0.001). In groups P-I and P-W, the no-liner subgroups showed significantly higher SBS values than the liner subgroups (*p* < 0.05). In group P-A, on the other hand, there was no significant difference in the value between the two subgroups (*p* = 0.461).

**Table 2:** Mean shear bond strength (MPa) (standard deviation) for the experimental groups (*n* = 10).

Group	No-Liner Subgroups	Liner Subgroups
Control	17.65 (2.98) Aa	26.99 (2.93) b
P-I	32.59 (3.58) Ba	27.80 (3.00) b
P-W	30.42 (2.27) Ba	27.84 (2.51) b
P-A	26.07 (1.93) Ca	26.78 (2.25) a

Within the no-liner subgroups, the same uppercase letter indicates lack of statistically significant difference among the groups (*p* > 0.05). Within the liner subgroups, one-way ANOVA revealed no significant differences in the values among the four groups (*p* = 0.742). For each row (each group), the same lowercase letter indicates lack of statistically significant difference between the two subgroups (*p* > 0.05).

**Figure 6:**
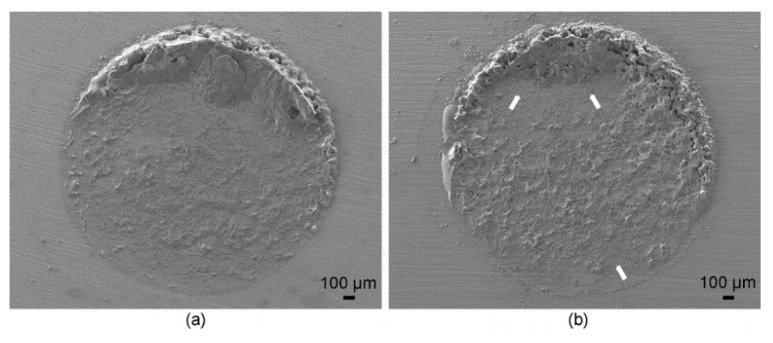
Representative SEM images of fractured zirconia sides (magnification: 37×) in the plasma-treated no-liner subgroups (**a**) and the liner subgroups (**b**). In Figure 6b, the white arrows represent complete delamination of porcelain or liner.

After SBS testing, all the veneered zirconia specimens exhibited a combination of cohesive and adhesive failure modes (*i.e.*, a mixed failure) (Figure 6). The fractures were found to start from the veneering porcelain and to propagate along the zirconia-porcelain interface. Within the no-liner subgroups, the control group showed primarily adhesive failure, whereas the three plasma-treated groups exhibited an increased area of cohesive failure (Figure 6a). In the four liner-applied groups, complete delamination of the porcelain or liner was frequently observed (Figure 6b).

## 3. Discussion

Zirconia surface is rather hydrophobic and has very low surface concentrations of –OH groups [18,19]. It has been reported that non-thermal atmospheric argon plasma treatment of any dental substrate can clean the surface and produce a super-hydrophilic surface (contact angle < 10°) [13,20]. In this *in vitro* study, the results of the surface characterization (Figure 2, Figure 3, Figure 4 and Figure 5, Table 1) indicate that the argon plasma treatment (group P-I) effectively cleaned the zirconia surfaces by reducing carbon-based contaminants and eventually created super-hydrophilic surfaces. Thus, the argon plasma treatment (group P-I) resulted in increased SBS between the zirconia and veneered porcelain in comparison with the control (untreated) group (Table 2), in accordance with the study by Canullo *et al.* [4]. Durability of surface properties after plasma treatment is also an important consideration in the practical application of this technology in a clinical setting [17]. When the veneering step was delayed for 24 h after plasma treatment, the water-storage condition (group P-W) maintained the properties of the cleaned, super-hydrophilic zirconia surfaces and did not significantly decrease the SBS. On the other hand, zirconia surfaces re-contaminated by being left in air (group P-A) underwent a significant decrease in SBS, although the value was still significantly higher than that of the control group. Thus, the null hypothesis that different storage conditions after plasma treatment do not affect the SBS between zirconia and veneered porcelain was rejected. In addition, liner application prior to veneering negatively affected the SBS values in groups P-I and P-W.

Previous studies showed that plasma surface treatment may alter the surface morphology [21,22]. In this study, a relatively long treatment time (600 s) and high power input (200 W) were employed during the plasma treatment. Nonetheless, the SEM and AFM examinations (Figure 2) clearly show that the plasma treatment used in this study did not induce much etching or destruction of the zirconia surfaces. The *R*_a_ values obtained in the AFM images also indicate no significant changes in surface topography. This finding may indicate great resistance of zirconia surface to plasma etching [23]. In addition, excited argon neutrals may have been less energetic than the high-energy electrons and ions, whose attack may cause surface damage [13].

Wettability and surface free energy of a surface are affected by both surface topography and physiochemistry [17]. Water CA reflects the wetting of the substrate by water and provides valuable information on the chemical composition of the substrate [13]. Despite only small changes in the surface morphology before and after the plasma treatment (Figure 2), the CA of zirconia (control: 71.0° ± 2.6°) was significantly reduced to 4.6° ± 1.0° in group P-I (Figure 3), which is close to a value for a super-hydrophilic surface (CA < 10°) [13,20]. A high CA indicates hydrophobic surface properties, and a dramatic drop in the CA may indicate the grafting of new polar functionalities onto the surface in addition to changes in chemical composition [13]. Thus, the reason for capability of the argon plasma treatment to reduce water CAs was examined using the XPS.

In the XPS analysis (Figure 4 and Figure 5, Table 1), decreased carbon (C) content was noted in the plasma-treated groups compared to the control, suggesting that organic matter was removed via breaking of C–C and C–H bonds [13]. In contrast, the atomic percent of oxygen (O) increased after the plasma treatment. In general, plasma treatment generates reactive species through bond-splitting induced by the vigorous atom/ion bombardment [13]. These reactive species can then bind to other atoms or functional groups. Non-thermal plasma is driven by argon gas, the oxygen from ambient air being excited to form highly reactive oxygen species, then to react with the surfaces [13,24]. The increase in oxygen-containing polar moieties on the surface enhances surface hydrophilicity [11]. A high O/C ratio indicates good wettability of the surface due to an increase in oxygen-containing groups [13,21,25]. The XPS analysis showed that the O/C ratios increased noticeably after plasma treatment (Table 1). This finding suggests the reason for the dramatic drop in the zirconia CA after plasma treatment (Figure 3). Thus, although the argon plasma treatment did not induce any new reactive functionality on the zirconia surface, it seems that treatment with inert gas effectively removed the organic contaminant on the surface through ion bombardment [26].

Maintaining plasma-induced surface properties is another consideration of practical importance. Although the veneering step was delayed for 24 h, simple immersion of the specimens in distilled water (group P-W) effectively maintained the super-hydrophilicity induced by plasma (CA: 5.6° ± 1.2°) (Figure 3). In addition, group P-W showed only a slight decrease in the O content and an increase in the C content, resulting in a decrease in the O/C ratio compared with group P-I (Table 1), suggesting that such water storage of plasma-treated specimens minimized surface re-contamination [17]. For the specimens left in air for 24 h (group P-A), in contrast, substantial “hydrophobic” recovery (CA: 20.7° ± 2.9°) and a much lower O/C ratio than those of groups P-I and P-W were observed, probably due to significant surface re-contamination with moisture, contaminants, or carbon dioxide in ambient air. These findings indicate that appropriate storage conditions that can minimize re-contamination are important in maintaining plasma-induced surface properties.

A more detailed study of the O 1s high resolution spectrum was performed. The O 1s peak was deconvoluted into three components, as shown in Figure 5. The first component at binding energy 530.2 eV can be assigned to ZrO_2_ (Zr–O–Zr), and the middle component at 531.8 eV to OH_acidic_ (acidic hydroxyl groups, Zr–O–H) [17,27]. The third component at 532.6 eV is assigned to OH_basic_ (basic hydroxyl groups, H–O–H) [17,27]. Among these, the third component can be ascribed to oxygen-containing impurities, such as water molecules, adsorbed on the surface [27,28]. The percentage area of the third component was greatest in the control group (36.07%) (Figure 5), indicating that cleaning only by ultrasonication (control) did not effectively remove the oxygen-containing contaminants from the specimens. As seen in the significant decrease in the third component in group P-I, oxygen-containing impurities were effectively removed from the zirconia surface by plasma cleaning. The third component of group P-W, similar to that of group P-I, suggests that most of the loosely bound water molecules were simply removed by a strong air blow after removing the specimen from the immersion water. The increased third component in group P-A compared to groups P-I and P-W indicates slight contamination (in particular the adsorption of water) in ambient air.

The SBS values for each group (Table 2) show that the bonding between zirconia and veneered porcelain was closely related to the surface properties of the zirconia specimens. Groups P-I and P-W showed superior zirconia-veneer bonding performance in comparison with the other groups, probably because the veneering porcelain wettability was effectively promoted on the clean, super-hydrophilic zirconia surfaces (Figure 3). In the failure pattern analysis, moreover, the control no-liner group showed primarily adhesive failure, whereas the three plasma-treated no-liner groups exhibited an increased area of cohesive failure (Figure 6a). However, a combination of plasma cleaning and liner application prior to veneering showed no positively synergistic effect on the bonding. The liner application was only positively effective for the untreated zirconia surfaces (control). When the liner was used after plasma cleaning, the SBS values significantly decreased in groups P-I and P-W (Table 2), which generally accords with the results of a recent previous study [4]. Moreover, complete delamination of the porcelain or liner was frequently observed in the liner-applied groups (Figure 6b), which is comparable to the finding of Kim *et al.* [5]. The substantially increased wettability in the two groups (Figure 3) may have caused a weakening in the liner bulk [4]. Moreover, it seems that great surface wettability of groups P-I and P-W put the veneering porcelain slurry in intimate contact with the zirconia surfaces without the intervention of a liner. Thus, simple plasma treatment seems to be a more effective and definite method than liner application for enhancing the bonding between zirconia and porcelain veneer. In addition, storage of plasma-treated specimens in water can be easily performed in dental laboratories and clinics without any additional equipment or facility. Long-term experimental and clinical investigations of plasma-treated zirconia-based restorations are still required to validate general clinical use of this relatively new technology. Moreover, this versatile technology may be effectively applied to various other dental bonding fields.

## 4. Materials and Methods

### 4.1. Zirconia Specimen Preparation

Rectangle-shaped (10 mm × 10 mm × 1 mm) sintered zirconia plates (KaVo Everest^®^ ZS-Ronde, Kaltenbach and Voigt GmbH and Co., Bismarcking, Germany) were prepared according to the manufacturer’s instructions. To produce a condition similar to that of milled zirconia, one surface (10 mm × 10 mm) of each specimen was ground with a polishing diamond disc (Allied High Tech Products, Rancho Domingues, CA, USA) under running water on a polishing machine (M-Prep3™, Allied High Tech Products) at a speed of 300 rpm [5]. Specimens were ultrasonically cleaned in acetone, alcohol, and distilled water for 10 min each, and finally air-dried. The average surface roughness *R*_a_ of each specimen was measured using a previously calibrated profilometer (Surftest SV-400, Mitutoyo Corp., Kawasaki, Japan) at a stylus speed of 0.1 mm/s, a cutoff of 0.8 mm, and a range of 600 mm and recorded as the average of the five readings [1]. The *R*_a_ of the zirconia specimens prepared using the polishing disc (0.233 ± 0.016 μm) was similar to that reported for milled zirconia (0.213 ± 0.002 μm) [5].

### 4.2. Plasma Treatment

**Figure 7:**
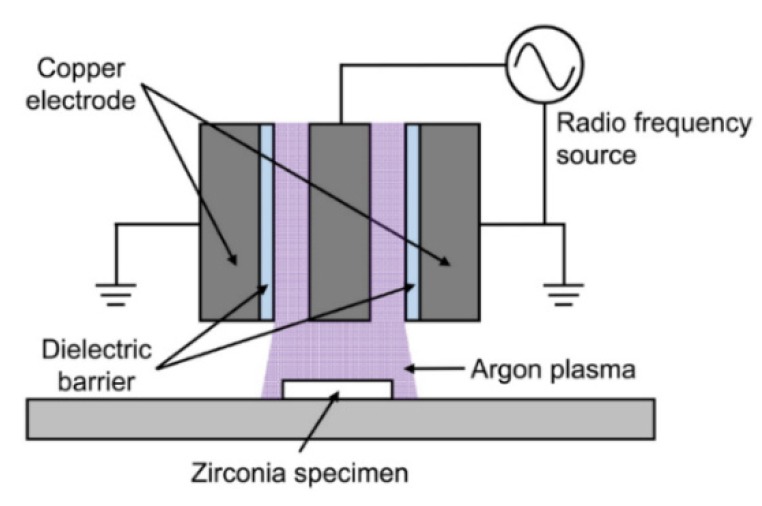
Schematic illustration of the atmospheric argon plasma system used in this study.

Non-plasma-treated (untreated) zirconia specimens were used as control. Except for the control group, all specimens were subjected to plasma treatment. A dielectric barrier discharge atmospheric argon plasma system (CJ08-P180, Changjo Engineering Co., Ltd., Daegu, Korea) with a 13.56 MHz radio frequency power supply was used (Figure 7). The plasma treatment on each zirconia specimen was performed at a discharge power of 200 W with argon for 600 s. The flow rate of argon was 20 slm. In group P-I, the plasma-treated specimens were immediately subjected to the subsequent porcelain veneering procedures. In group P-W, the specimens were stored in distilled water in a plastic container for 24 h, and the surfaces then dried using a strong air blow to ensure the complete removal of residual water from the surfaces prior to veneering procedures. In group P-A, the specimens were left in air for 24 h and then subjected to veneering procedures. The storage was performed in a temperature controlled room at 23 °C ± 1 °C with relative humidity at 50% ± 5%.

### 4.3. Surface Examinations

The untreated (control) and plasma-treated zirconia surfaces were observed by a field emission-scanning electron microscope (FE-SEM, JSM-6700F, Jeol, Tokyo, Japan). Specimens were sputter-coated with platinum and photographs of representative areas of the surfaces were taken at 2000× magnification.

In addition, the untreated and treated zirconia surfaces were examined by atomic force microscopy (AFM, XE-100, Park Systems Corp., Suwon, Korea). During analysis, the microscope was operated in non-contact mode with a Si_3_N_4_ V-shaped cantilever (*k* = 42 N/m). Images with 256 × 256 pixels were taken in air with a scan size of 5 μm × 5 μm and a scan rate of 0.5 Hz. Three measurements were performed for each specimen using a standardized rectangular spot (1.5 μm × 1.5 μm). The *R*_a_ of each specimen was recorded as the average of the three readings (*n* = 6).

### 4.4. Contact Angle Measurements

To compare the zirconia surface wettability, the contact angles (CAs) of water droplets on the surfaces were measured (using the static sessile drop method) by a surface goniometer (OCA 15 plus, Data-Physics Instrument GmbH, Filderstadt, Germany) (*n* = 6). CA measurements were performed in a temperature-controlled room at 23 °C ± 1 °C with relative humidity at 50% ± 5%.

### 4.5. X-ray Photoelectron Spectroscopy

The chemical compositions of the outermost surfaces of zirconia specimens were analyzed using X-ray photoelectron spectroscopy (XPS, K-Alpha, Thermo Fisher Scientific, East Grinstead, UK) with a monochromatic Al Kα X-ray source. Pass energies were 200 and 20 eV for survey scans and high resolution scans, respectively. The pressure within the XPS chamber was 10^−7^ to 10^−8^ Pa. Photo-emitted electrons were collected at a take-off angle of 90°. The intensities of Zr 3d, O 1s, Y 3d, and C ls were determined. Deconvolution of the O 1s core-level spectra was performed with a mixed Gaussian/Lorentzian model after a Shirley-type background subtraction [29]. Data acquisition and processing were performed using Thermo Advantage software (Thermo Fisher Scientific).

### 4.6. Veneering Procedures

Eighty rectangle-shaped (10 mm × 10 mm × 1 mm) sintered zirconia plates were prepared and embedded “loosely” in round silicone rubber molds using an acrylic resin. The embedded zirconia specimens were divided into four groups (control, groups P-I, P-W, and P-A), each group then being further separated into two subgroups according to the use of liner prior to veneering (no-liner and liner subgroups) (*n* = 10). For the plasma-treated groups, the zirconia plates were temporarily removed from the embedding resin during the plasma treatment.

**Figure 8:**
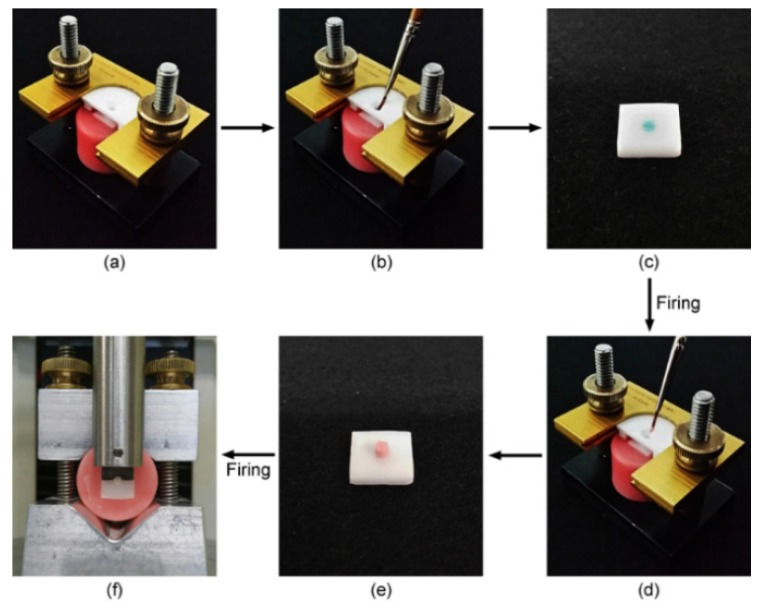
Porcelain veneering and subsequent shear bond strength testing procedures using Ultradent jig. (**a**) Zirconia specimen placed into the jig; (**b**) Liner application; (**c**) Liner applied to the zirconia surface; (**d**) Veneering porcelain on the liner; (**e**) Porcelain veneered on the surface; (**f**) Shear bond strength testing. In the no-liner subgroups, steps (b,c) were omitted.

The veneering porcelain onto the zirconia surfaces was performed using an Ultradent jig (Ultradent Products Inc., South Jordan, UT, USA), as shown in Figure 8. A loosely embedded zirconia specimen was placed into the jig and the surface to be veneered was isolated using a cylindrical-shaped plastic matrix (internal diameter: 2.38 mm). In the liner subgroups, a thin coat of a ceramic liner paste (Zr-Adhesive, Heraeus Kulzer GmbH, Hanau, Germany) was applied onto the zirconia surface through the matrix. The liner-coated zirconia plate was removed from the jig and the embedding resin and then subjected to the firing procedure in a porcelain furnace (Vacumat 40, Vita Zahnfabrik, Bad Säckingen, Germany). A mixed porcelain slurry (Heraceram Zirconia, Heraeus Kulzer GmbH) was applied onto the liner region through the matrix. After excess liquid was blotted with absorbent paper, the veneered zirconia plate was removed from the jig and the embedding resin, then subjected to the firing procedure in the Vacumat 40 porcelain furnace. In the no-liner subgroups, a mixed porcelain slurry was directly applied to the zirconia surface without the above liner application procedures. The firing temperature/holding time was 1050 °C/10 min and 870 °C/1 min for the liner and the veneering porcelain, respectively, following the manufacturer’s instructions.

### 4.7. Shear Bond Strength Testing

Prior to shear bond strength (SBS) testing, the veneered specimen was firmly fixed in the embedding resin by adding additional acrylic resin around the specimen (Figure 8f). Specimens were then perpendicularly engaged at their veneered porcelain cylinder bases with a round-notched custom shear blade (Ultradent Products Inc.) in a universal testing machine (Model 3343, Instron Inc., Canton, MA, USA) at a crosshead speed of 1.0 mm/min until bonding failure occurred (Figure 8f). Bond strengths (MPa) were calculated from the peak load of failure (N) divided by the veneered surface area, the diameter of which (~2.38 mm) for each specimen had been measured using a vernier caliper prior to SBS testing. Following debonding, all fractured interfaces were examined under an optical microscope (SZ61, Olympus, Tokyo, Japan) at 20× magnification to determine the mode of failure. These were classified into one of the three types: adhesive failure at the zirconia-veneer interface; cohesive failure within the veneered porcelain; and a combination of these two failure modes (mixed failure).

### 4.8. Statistical Analysis

All data were examined for the normality of their distribution (Shapiro-Wilk test) and for the equality of the variances (Levene test). The *R*_a_ of the zirconia surfaces before and after the plasma treatment was statistically compared by Student’s *t* test. The CA values of the four groups were compared using one-way analysis of variance (ANOVA) followed by Tukey’s *post hoc* test. For the SBS data, two-way ANOVA was used to test the main effects of the storage conditions (including control) and the use/non-use of the liner. Based on the results from the initial analysis, the data file was stratified and analyzed using one-way ANOVA followed by Tukey’s *post hoc* test or Student’s *t* test to determine whether the differences in SBS were statistically significant [30]. All statistical analyses were carried out using SPSS 17.0 for Windows (SPSS Inc., Chicago, IL, USA) at a level of significance of α = 0.05.

## 5. Conclusions

The present *in vitro* study suggests that non-thermal atmospheric argon plasma treatment of zirconia effectively produced a clean, super-hydrophilic surface, which seems appropriate for the optimal wetting of veneering porcelain and, as a result, significantly enhanced the bonding between the zirconia and veneering porcelain. The plasma-induced super-hydrophilicity was effectively maintained by simple water immersion of the specimens until porcelain veneering.

## References

[1] Kim M.J., Kim Y.K., Kim K.H., Kwon T.Y. (2011). Shear bond strengths of various luting cements to zirconia ceramic: Surface chemical aspects. J. Dent..

[2] Janyavula S., Lawson N., Cakir D., Beck P., Ramp L.C., Burgess J.O. (2013). The wear of polished and glazed zirconia against enamel. J. Prosthet. Dent..

[3] Chaiyabutr Y., McGowan S., Phillips K.M., Kois J.C., Giordano R.A. (2008). The effect of hydrofluoric acid surface treatment and bond strength of a zirconia veneering ceramic. J. Prosthet. Dent..

[4] Canullo L., Micarelli C., Bettazzoni L., Magnelli A., Baldissara P. (2014). Shear bond strength of veneering porcelain to zirconia after argon plasma treatment. Int. J. Prosthodont..

[5] Kim H.J., Lim H.P., Park Y.J., Vang M.S. (2011). Effect of zirconia surface treatments on the shear bond strength of veneering ceramic. J. Prosthet. Dent..

[6] Sailer I., Feher A., Filser F., Gauckler L.J., Luthy H., Hammerle C.H. (2007). Five-year clinical results of zirconia frameworks for posterior fixed partial dentures. Int. J. Prosthodont..

[7] Wang G., Zhang S., Bian C., Kong H. (2014). Interface toughness of a zirconia-veneer system and the effect of a liner application. J. Prosthet. Dent..

[8] Choi Y.S., Kim S.H., Lee J.B., Han J.S., Yeo I.S. (2012). *In vitro* evaluation of fracture strength of zirconia restoration veneered with various ceramic materials. J. Adv. Prosthodont..

[9] Schmitt J., Goellner M., Lohbauer U., Wichmann M., Reich S. (2012). Zirconia posterior fixed partial dentures: 5-year clinical results of a prospective clinical trial. Int. J. Prosthodont..

[10] Tarumi N., Uo M., Yamaga E., Watari F. (2012). SEM observation and wettability of variously processed and fractured surface of dental zirconia. Appl. Surf. Sci..

[11] Wang G., Zhang S., Bian C., Kong H. (2014). Effect of zirconia surface treatment on zirconia/veneer interfacial toughness evaluated by fracture mechanics method. J. Dent..

[12] Ritts A.C., Li H., Yu Q., Xu C., Yao X., Hong L., Wang Y. (2010). Dentin surface treatment using a non-thermal argon plasma brush for interfacial bonding improvement in composite restoration. Eur. J. Oral Sci..

[13] Chen M., Zhang Y., Sky Driver M., Caruso A.N., Yu Q., Wang Y. (2013). Surface modification of several dental substrates by non-thermal, atmospheric plasma brush. Dent. Mater..

[14] Rodríguez-Villanueva C., Encinas N., Abenojar J., Martínez M.A. (2013). Assessment of atmospheric plasma treatment cleaning effect on steel surfaces. Surf. Coat. Technol..

[15] Liebermann A., Keul C., Bahr N., Edelhoff D., Eichberger M., Roos M., Stawarczyk B. (2013). Impact of plasma treatment of PMMA-based CAD/CAM blanks on surface properties as well as on adhesion to self-adhesive resin composite cements. Dent. Mater..

[16] Chan Y.H., Kim J.K., Liu D., Liu P.C.K., Cheung Y.M., Ng M.W. (2005). Effect of plasma treatment of Au-Ni-Cu bond pads on process windows of Au wire bonding. IEEE Trans. Adv. Packag..

[17] Noro A., Kaneko M., Murata I., Yoshinari M. (2013). Influence of surface topography and surface physicochemistry on wettability of zirconia (tetragonal zirconia polycrystal). J. Biomed. Mater. Res. B.

[18] Lohbauer U., Zipperle M., Rischka K., Petschelt A., Müller F.A. (2008). Hydroxylation of dental zirconia surfaces: Characterization and bonding potential. J. Biomed. Mater. Res. B..

[19] Piascik J.R., Swift E.J., Braswell K., Stoner B.R. (2012). Surface fluorination of zirconia: Adhesive bond strength comparison to commercial primers. Dent. Mater..

[20] Nam Y., Sharratt S., Byon C., Kim S.J., Ju Y.S. (2010). Fabrication and characterization of the capillary performance of superhydrophilic Cu micropost arrays. J. Microelectromech. Syst..

[21] Yaman N., Özdoğan E., Seventekin N. (2011). Atmospheric plasma treatment of polypropylene fabric for improved dyeability with insoluble textile dyestuff. Fibers Polym..

[22] Da Silva M.A.M., Guerra Neto C.L.B., Nunes Filho A., Freitas D.O., Braz D.C., Alves C. (2013). Influence of topography on plasma treated titanium surface wettability. Surf. Coat. Technol..

[23] Choi Y.R., Kwon J.S., Song D.H., Choi E.H., Lee Y.K., Kim K.N., Kim K.M. (2013). Surface modification of biphasic calcium phosphate scaffolds by non-thermal atmospheric pressure nitrogen and air plasma treatment for improving osteoblast attachment and proliferation. Thin Solid Films.

[24] Fricke K., Steffen H., von Woedtke T., Schroder K., Weltmann K.D. (2011). High rate etching of polymers by means of an atmospheric pressure plasma jet. Plasma Process. Polym..

[25] Sun S., Sun J., Yao L., Qiu Y. (2011). Wettability and sizing property improvement of raw cotton yarns treated with He/O_2_ atmospheric pressure plasma jet. Appl. Surf. Sci..

[26] Yavirach P., Chaijareenont P., Boonyawan D., Pattamapun K., Tunma S., Takahashi H., Arksornnukit M. (2009). Effects of plasma treatment on the shear bond strength between fiber-reinforced composite posts and resin composite for core build-up. Dent. Mater. J..

[27] Mi Y., Wang J., Yang Z., Wang Z., Wang H., Yang S. (2014). A simple one-step solution deposition process for constructing high-performance amorphous zirconium oxide thin film. RSC Adv..

[28] Homola T., Matoušek J., Medvecká V., Zahoranová A., Kormunda M., Kováčik D., Černák M. (2012). Atmospheric pressure diffuse plasma in ambient air for ITO surface cleaning. Appl. Surf. Sci..

[29] Piascik J.R., Wolter S.D., Stoner B.R. (2011). Development of a novel surface modification for improved bonding to zirconia. Dent. Mater..

[30] Maryanchik I., Brendlinger E.J., Fallis D.W., Vandewalle K.S. (2010). Shear bond strength of orthodontic brackets bonded to various esthetic pontic materials. Am. J. Orthod. Dentofac. Orthop..

